# Co-modulation of Liver Genes and Intestinal Microbiome of Largemouth Bass Larvae (*Micropterus salmoides*) During Weaning

**DOI:** 10.3389/fmicb.2020.01332

**Published:** 2020-06-17

**Authors:** Liulan Zhao, Kuo He, Jie Luo, Junlong Sun, Lei Liao, Xiaohong Tang, Qiao Liu, Song Yang

**Affiliations:** College of Animal Science and Technology, Sichuan Agricultural University, Chengdu, China

**Keywords:** largemouth bass, weaning, larvae, transcriptome, intestinal microbes, intestinal digestive enzyme

## Abstract

In recent years, largemouth bass have become one of the most commonly aquacultured species in China, however, its low survival rate during larval weaning has always been a bottleneck that has restricted industrial development. Understanding the changes in liver metabolism and intestinal microflora during the weaning of largemouth bass larvae can help to design better weaning strategies and improve survival. In this study, liver mRNA and intestinal microflora 16S rRNA genes were analyzed using high-throughput sequencing at the pre, mid, and post weaning stages [15, 30, 45 days post hatching; total length (cm) were 2.21 ± 0.12, 3.45 ± 0.21, 5.29 ± 0.33, respectively]. The transcriptome results revealed that the genes with increased expression were related to amino acid metabolism in the pre-weaning stage, but they were related to fatty acid metabolism in the post-weaning stage. A similar phenomenon was observed in the intestinal microflora where the dominant microbe Proteobacteria (relative abundance 56.32%) in the pre-weaning stage was gradually replaced by Firmicutes (relative abundance 62.81%) by the post-weaning stage. In addition, the three most important digestive enzymes (trypsin, lipase, and amylase) in the intestine were significantly decreased during the mid-weaning stage (*P* < 0.05), which was also true for some genes crucial to immune pathways in the liver. Overall, these findings showed that weaning in largemouth bass can cause changes in liver metabolism and intestinal microbial communities, which has improved our understanding of fish adaptation to changes in food sources during weaning.

## Introduction

While the global demand for aquatic products strongly increased over the last 20 years, fisheries resources have sharply declined due to overfishing, prompting the aquaculture industry to rapidly increase its production, making it the fastest growing sector in the animal production field (FAO, 2018). However, many fishes have difficulty transitioning from live prey to formulated diets (weaning) as they mature through their larval stages, a transition that has consistently proven to be a major bottleneck in fish farming (Rønnestad et al., 2013; Sun et al., 2015). Weaning in aquaculture is the process of gradually replacing live prey with artificial feed in fish larvae. Weaning has been investigated in a variety of fishes, with a focus on the effects of the co-feeding method, start time, and duration of weaning on growth performance and survival of larvae. It can be observed that different weaning strategies should be adopted for different species, pikeperch larvae, for example, can be weaned from day 15 dph (days post hatching) (Hamza et al., 2007) while sea bass larvae should be weaned at 20 dph (Suzer et al., 2007). In addition, the success rate of weaning can be affected by the nutrient composition of formula feeds (Gisbert and Mozanzadeh, 2019).

As a matter of fact, the larvae of most fish exhibit better growth performance and survival when fed with live prey. Nonetheless, the use of inert micro-diets to replace live feed during early developmental stages has been increasing due to its obvious benefits to the farming of this species. Namely, the inert micro-diets provide consistent nutrient feed that is ready-to-use with little preparation time, whereas live prey may act as vectors for diseases, have variable nutritional value that is difficult to manipulate, have high production costs, and a time consuming preparation process (Hart and Purser, 1996; Hamza et al., 2007; Pinto et al., 2018). Therefore, further investigations into weaning are of great significance for the optimization of aquaculture of any farmed species.

In recent years, there has been increasing interest in gastrointestinal (GI) microbiota of animals as they play an indispensable roles in in their host’s nutrient metabolism, growth and development, immunity, and resistance to invading pathogens (Wang et al., 2018). GI microbiota are in a constant state of dynamic change in an animal’s gut. In fish, it is well known that GI microbiota are affected by a range of factors, including host characteristics (e.g., genetics, age, immunity, and intestinal motility) (Li et al., 2012; Navarrete et al., 2012; Stephens et al., 2016) environmental factors (e.g., water, diet, and medicine/antibiotics) (Sullam et al., 2012; Ringø et al., 2016; Dehler et al., 2017) and microbial factors (e.g., adhesion capacity, enzymes, and metabolic capacity) (Prakash et al., 2011). Weaning can lead to changes in the intestinal microbiota of fish, but little is known about how and why these changes occur. However, with the advent of next-generation sequencing (NGS) technologies and the development of various bioinformatics tools like 16S rRNA sequencing, the study of microbial communities has been greatly advanced (Ghanbari et al., 2015). NGS has also made the analysis of transcriptomes more efficient, reliable, comprehensive, and affordable, making a powerful tool more easily accessible and enabling the better understanding potential pathways that control host cell fate, metabolism, development, and disease progression. This technique has even enabled the exploration of the transcriptomes of non-model organisms for which reference genomes are not available (Mutz et al., 2013; Sudhagar et al., 2018).

The largemouth bass (*Micropterus salmoides*) was introduced into China in 1983 from North America for the purpose of establishing a staple aquaculture species. Its fast growth rate, wide temperature tolerance, adaptive plasticity to new environments, and appeal as a food source led it to becoming one of China’s primary aquaculture fish species, with annual production of up to 450,000 tons (Bai et al., 2008; China Statistical Yearbook, 2018). However, even though great breakthroughs on the use of artificial compound feed in largemouth bass aquaculture have been made, the low success rate during weaning remains a bottleneck in the process of largemouth bass farming. This transition has not been significantly improved upon due to a generally poor understanding based on a limited number of basic studies, meaning that larval weaning mortality has remained high along with production costs. Thus, the specific objective of this study was to comprehensively investigate the characteristics of the liver transcriptome and intestinal microflora in largemouth bass larvae during weaning. The goal of this study was to contribute to a better understanding of the weaning process in largemouth bass, and also provide a theoretical basis and reference for the weaning of largemouth bass larvae.

## Materials and Methods

### Larval Rearing and Sampling

For this experiment, 200 thousand yolk-sac larvae were obtained from a commercial farm in Panzhihua city (Sichuan, China) and reared in a larval rearing pond (60 m × 30 m × 1.2 m) at the Dongpo commercial farm (Meishan, Sichuan, China). During the rearing period, the water quality parameters were as follows: temperature was 27.8–31.2°C; dissolved oxygen was 6.3–7.7 mgml^–1^; pH was 7.6–8.2; ammonia nitrogen was below 0.1 mg/L; and nitrite was below 0.01 mg/L. Larvae reared in larval rearing pond fed on the zooplankton in the pond (rotifers and Cladocerans) from 3 to 7 dph (days post hatching). Subsequently, larvae were fed with a mixed diet of zooplankton and *Chironomus plumosus* up to 15 dph, because the zooplanktons of the pond were no longer sufficient to sustain the larvae.

The weaning operation was carried out in a large pond (60 m × 30 m × 1.2 m), and the individuals were used for replicates. During the weaning program, the larvae were subjected to co-feeding with *Chironomus plumosus* (42% ∼ 62% crude protein, 2% ∼ 8% crude lipid) and a commercial larval micro-diet (52% crude protein, 4% crude lipid, 4% crude fiber) where the proportion of commercial micro diet was increased slowly until 35 dph, at which time fish were fed entirely with the commercial larval micro-diet until to the end of the experiment. The larvae were fed three times a day at 7:00, 12:00, and 17:00. Feeding time lasted more than an hour to feed the bait little by little, because larvae weaning required finesse and patience to lure the larvae consuming micro-diet. In addition, a semi-automatization device was used to attract the fry to concentrate at the feeding site during weaning stages, which could slowly spray water mixed with *Chironomus plumosus.*

Samples were collected randomly before feeding in the morning from the pond on 15, 30, and 45 dph, representing pre-weaning, mid-weaning, and post-weaning fries, respectively. The larvae body weight (g) were 0.103 ± 0.01, 0.47 ± 0.10, 1.74 ± 0.33 in the three weaning stages of this study, and total length (cm) were 2.21 ± 0.12, 3.45 ± 0.21, 5.29 ± 0.33, respectively (Table 1). Liver and gut tissues were rapidly dissected out of the 120 experimental fish fries at each sampling point, and frozen immediately in liquid nitrogen and stored in a −80°C freezer until further analyses, which included transcriptome, microbiome, and enzymatic determination.

**TABLE 1:** The growth data of largemouth bass larvae in each weaning stage.

Weaning stage	Body weight (g)	Body length (cm)	Total length (cm)	Average of growth/d		
				Body weight (g)	Body length (cm)	Total length (cm)
Pre	0.103 ± 0.01	1.84 ± 0.09	2.21 ± 0.12			
				0.024	0.074	0.082
Mid	0.47 ± 0.10	2.95 ± 0.25	3.45 ± 0.21			
				0.085	0.102	0.123
Post	1.74 ± 0.33	4.48 ± 0.30	5.29 ± 0.33			

### Liver Transcript Sequencing

Since the larval samples were very small, 10 fish livers were pooled into a single sample, and a total of three samples were collected for transcriptome analysis at each sampling time. The total RNA was extracted from livers with the Animal Total RNA Isolation Kit (Foregene, Chengdu, China) according to the manufacturer’s instructions. RNA was quantified and quality was assessed using the NanoDrop Spectrophotometer 2000c (NanoDrop Technologies, Wilmington, DE, United States) and 2100 Bioanalyzer (Agilent Technologies, Palo Alto, CA, United States). The RNA of three samples from each group were used for library construction, where optical density (OD) ratios of 260/280 and 260/230 were greater than 1.8 and the RNA integrity number (RIN) was greater than 8.

The qualified libraries were Paired-end sequenced using the BGISEQ-500 system. Meaningless data were removed, this included low-quality reads and adaptor-polluted reads. The Q20 and Q30 of the clean data were calculated, and all downstream analyses were performed using the clean, high-quality data. Trinity^1^ (version: v2.0.6) (Grabherr et al., 2011) was used to perform de novo assembly with clean reads, followed by Tgicl (see footnote 1, version: v2.0.6) (Pertea et al., 2003) to cluster transcripts into unigenes. After assembly, unigenes were used for functional annotation through comparisons with the NR, NT, KOG, SwissProt, and InterPro databases. Expression of genes was quantified using the DESeq package, and differentially expressed genes (DEGs) among the stages were identified using the following criteria: fold change ≥ 1.5 and adjusted *q*-value ≤ 0.05. The goal of DEGs analysis was to determine if gene expression in one stage was significantly different from the other two stages. Kyoto Encyclopedia of Genes and Genomes (KEGG) pathway analyses were then carried out with the differentially expressed genes of each stage as input.

To validate the reliability of the data obtained by RNA-seq, SYBR Green Real-time RT-PCRs were performed on a CFX Connect Real-Time system (Bio-Rad Laboratories, United States) using the qRT-PCR Detection Kit (Takara, Dalian, China). β-actin was used as an internal control, with three biological and three technical replicates. The target gene qRT-PCR primers were designed with reference to the transcriptome sequences using Primer 5.0, the primer sequences are listed in Supplementary Table S1. The 2^–ΔΔCt^ method was used to calculate relative expression levels (Livak and Schmittgen, 2001).

### Intestinal Digestive Enzymes

The intestines of five fish fries were pooled to make one sample, and a total four samples were collected at each stage to determine digestive enzymatic activity. All the samples were homogenized in 2 mL of chilled PBS buffer on ice, and then centrifuged at 3,000 × *g* for 10 min at 4°C to obtain a clear supernatant (3–18 K, Sigma^®^, Germany). Finally, the clear supernatant was used for quantitative assays of trypsin, lipase, and amylase activities, and total protein. The enzyme activities and total protein were measured by detection kits purchased from the Nanjing Jiancheng Bioengineering Institute (Nanjing, China), which included trypsin, lipase, amylase, and total protein assay kits (Product codes: A080-2-2, A054-1-1, C016-1-1, and A045-3-2, respectively).

### Intestinal Microbial 16S rRNA Sequencing

The intestines of five fish fries were pooled in one sample, and a total 10 samples were collected at each stage for microbial community analysis. Total genomic DNA was isolated from gut tissue using the TIANamp Stool DNA Kit according to the manufacturer’s instructions (Tiangen Biotech (Beijing) CO., LTD. China). Concentrations of extracted DNA were determined using the NanoDrop Spectrophotometer 2000c (NanoDrop Technologies, Wilmington, DE, United States). 16S rRNA genes from distinct regions (16S V3-V4) were amplified using specific primers (341F 5′-CCTAYGGGRBGCASCAG-3′ and 806R 5′-GGACTACNNGGGTATCTAAT-3′). All PCR reactions were carried out in 30 μL reaction buffer with 15 μL of Phusion^®^ High-Fidelity PCR Master Mix (New England Biolabs), 0.2 μM of forward and reverse primers, and about 10 ng template DNA. Thermal cycling consisted of initial denaturation at 98°C for 1 min, followed by 30 cycles of denaturation at 98°C for 10 s, annealing at 50°C for 30 s, and elongation at 72°C for 30 s. Finally, the reaction was held at 72°C for 5 min. Equal volumes of 1 × loading buffer (contained SYB green) and PCR products were subjected to 2% agarose gel electrophoresis for detection. PCR products were mixed in equidensity ratios. Then, the mixture of PCR products was purified with GeneJETTM Gel Extraction Kit (Thermo Scientific). Sequencing libraries were generated using Ion Plus Fragment Library Kit 48 rxns (Thermo Scientific) following the manufacturer’s recommendations. The library quality was assessed on the Qubit@ 2.0 Fluorometer (Thermo Scientific), and finally, the library was sequenced on an Ion S5TM XL platform and 400 bp/600 bp single-end reads were generated.

The USEARCH software was used to filter the raw reads based on quality, removing chimeric sequences and non-bacterial sequences, cluster reads with similarities ≥ 97% were used as operational taxonomic units (OTUs), and to assemble an OTU table. Then, representative sequences of the most abundant sequences from the OTUs were classified and annotated by the RDP classifier classification algorithm and compared with the database. The representative OTU sequences were classified to the genus level with a threshold of 0.8, and the alpha and beta diversity analyses were performed.

### Statistical Analysis

An intergroup correlation analysis (ICA) was performed between the matrices of the microbiome relative abundance table and the transcriptomic data using the OmicShare ICA tools^2^. SPSS Statistics 22.0 software (SPSS, Chicago, IL, United States) was used to analyze the results of digestive enzyme activity and qRT-PCR (SPSS, Chicago, IL, United States) applying a one-way analysis of variance, followed by Fisher’s least significant difference *post hoc* test and Duncan’s multiple range tests. A *P*-value of <0.05 was considered statistically significant. Data were presented as means ± standard error.

## Results

### Liver Transcriptome

#### Brief Description of Liver RNA-seq Data

A total of 9 cDNA libraries were sequenced from the liver samples of the three weaning stages (*n* = 3 per stage). After removing adaptors and filtering, sequencing generated an average of 65.99M clean reads from 9 samples at three stages using the BGISEQ-500 platform. After assembly and redundancy, 42,631 unigenes were obtained. The total length, average length, N50, and GC contents were 62,787,387 bp, 1,472 bp, 2,659 bp, and 45.03%, respectively (Supplementary Tables S2, S3). Then, unigenes were compared to the seven functional databases for annotation, which ended up with 29,423 (NR: 69.02%), 34,991 (NT: 82.08%), 25,705 (SwissProt: 60.30%), 22,957 (KOG: 53.85%), 26,035 (KEGG: 61.07%), 6,147 (GO: 14.42%) and 21,223 (Pfam: 49.78%) unigene functional annotations being obtained. Nearly 70% of the species annotated by Nr were in the order Perciformes, including *Larimichthys crocea* (24.33%) and *Lates calcarifer* (27.74%).

#### Differentially Expressed Genes Among the Three Stages

**FIGURE 1:**
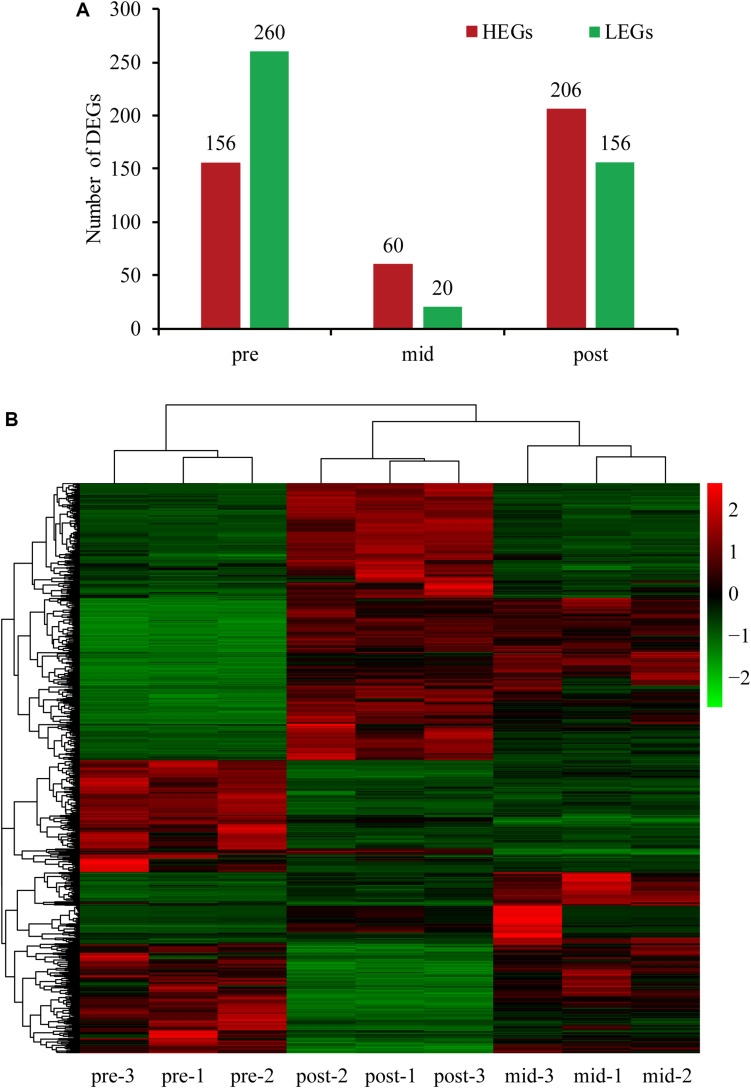
**(A)** Number of DEGs in the three weaning stages. **(B)** Heatmap of the DEGs among three stages. HEGs, higher expression genes; LEGs, lower expression genes.

Differentially expressed genes, including both increased and decreased expression, were defined as genes with FPKM (reads per kilo base of exon model per million mapped reads) log_2_-transformed fold changes >1.5 or < −1.5, compared to the other two stages (*q* < 0.05). In total, there were 156 genes with increased expression and 260 with decreased expression in the pre-weaning stage, 60 genes with increased expression and 20 with decreased expression in the mid-weaning stage, and 206 genes with increased expression and 165 with decreased expression in the post-weaning stage (Figure 1A). In order to more intuitively understand the differential gene-specific expression patterns of the three stages, the DEGs were hierarchically clustered as shown in Figure 1B.

#### Pathway Analysis of Differentially Expressed Genes

**FIGURE 2:**
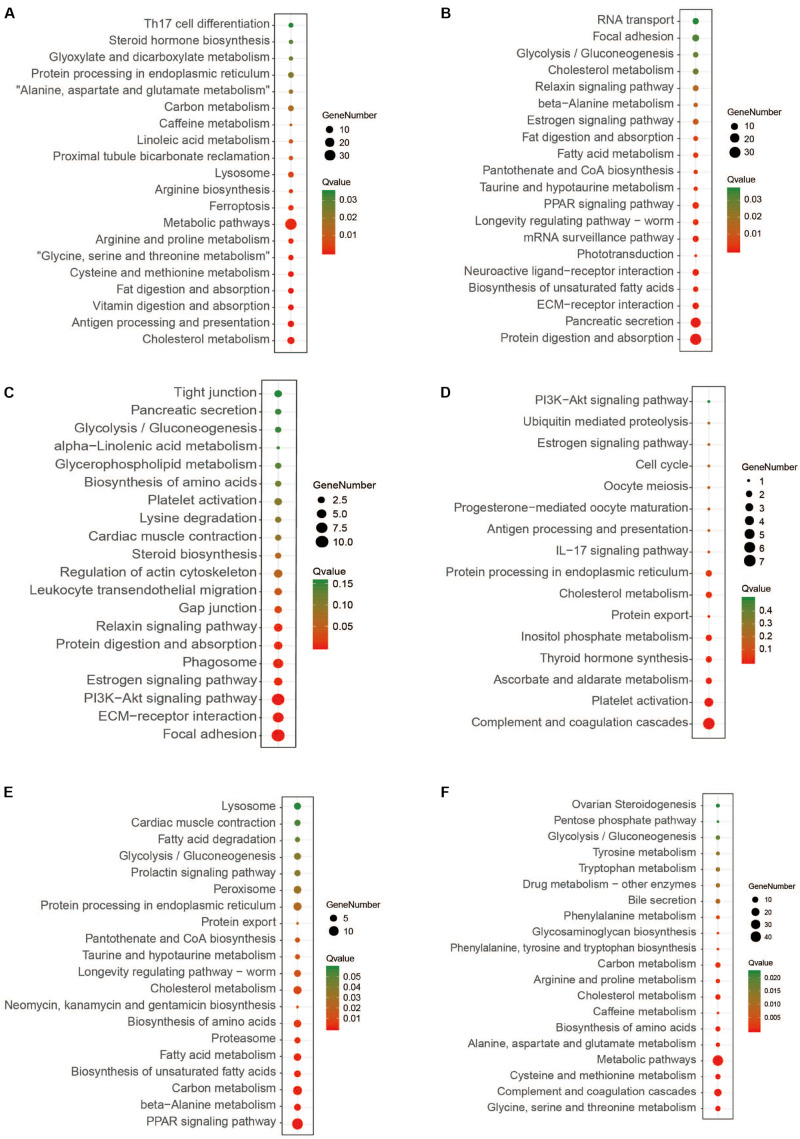
The top 20 KEGG enrichment pathways of the three stages. **(A,C,E)** HEGs in pre, mid, and post-weaning stages, **(B,D,F)** LEGs in pre, mid, and post-weaning stages, respectively. HEGs, higher expression genes; LEGs, lower expression genes.

To explore the putative functional biochemical pathways of the DEGs, KEGG (Kyoto Encyclopedia of Genes and Genomes) enrichment analyses were performed for all stages. The top 20 enriched pathways with the smallest q values are displayed in scatter plots in Figure 2, and the main enrichment pathways are shown in the Supplementary Table S4 (the unigenes are shown in Supplementary Data Sheet S1). In the pre-weaning stage, the enriched pathways based on the genes with increased expression were related to amino acid biosynthesis and metabolism, and the enriched pathways based on the genes with decreased expression were related to fatty acid digestion, absorption, and metabolism. Interestingly, the pathways enriched in post-weaning stage seemed to be opposite those of the pre-weaning stage, that is to say, the pathways related to fatty acid biosynthesis and metabolism were enriched by the genes with increased expression and the genes with decreased expression enriched pathways related to amino acid biosynthesis and metabolism in the post-weaning stage. Finally, in the mid-weaning stage, the genes with increased expression were enriched in the pathways related to environmental information processing and cellular processes, while the genes with decreased expression were enriched in the pathways related to the immune system, such as complement and coagulation cascades, platelet activation, and the IL-17 signaling pathway.

#### Confirmation of DEGs by qRT-PCR

In order to confirm the RNA-seq data, nine DEGs were selected for qRT-PCR validation. In general, the expression patters of all nine selected DEGs identified by qRT-PCR were similar to those from the RNA-seq analysis. While the relative expression levels were not perfectly consistent, the results do confirm the reliability and accuracy of the RNA-seq data (Supplementary Figure S1).

### Intestinal Microbes

#### 16S rRNA Sequencing Data, OTU Diversity, and Similarity Analyses

**FIGURE 3:**
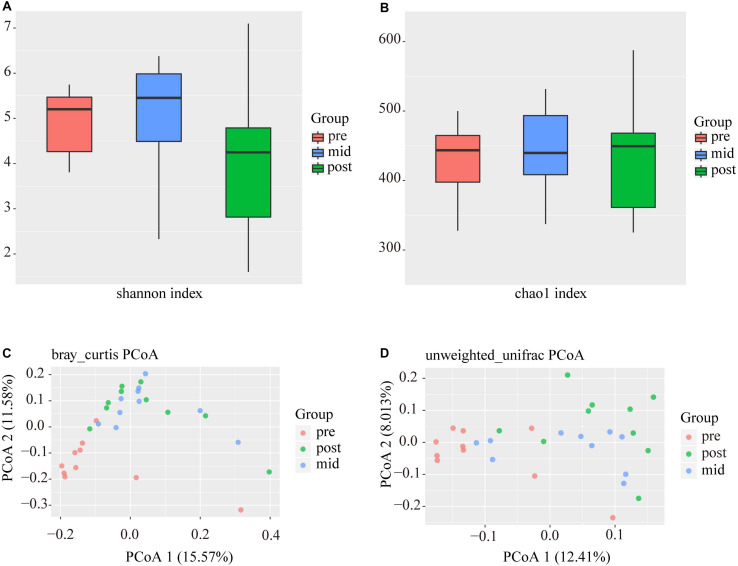
Alpha and beta-diversity analyses of intestinal bacterial OTUs of the three stages. **(A)** and **(B)** are Shannon diversity index and Chao 1 index of intestinal bacterial OTUs of the three stages. **(C)** and **(D)** are Principal coordinate analysis (PCoA) based on Bray-Curtis and Unweighted-Unifrac distance of intestinal bacterial OTUs of the three stages, respectively.

After data filtering, quality control, the removal of primers, chimeras, and low confidence singletons, a total of 2,046,395 V3-V4 16S rRNA sequence reads from the 30 samples, an average of 68,213 sequence reads from each sample, were used in this study. The overall number of OTUs detected by our analysis reached 5123 based on 97% nucleotide sequence similarity between reads. Through comparisons with the database 16S sequences, 28 phyla and 290 genera were annotated, using a threshold of 0.8. In this study, the Chao and Shannon indexes were used to calculate the alpha-diversity of the three stages (Figures 3A,B), which did not differ significantly in any case. Beta diversity analyses were performed using the Bray–Curtis similarity metric and unweighted-unifrac PCoA and revealed that the samples clustered according to stage (Figures 3C,D), where it was found that some similarities existed between the mid and post-weaning stages.

#### Taxonomic Composition of Intestinal Microbial Communities

**FIGURE 4:**
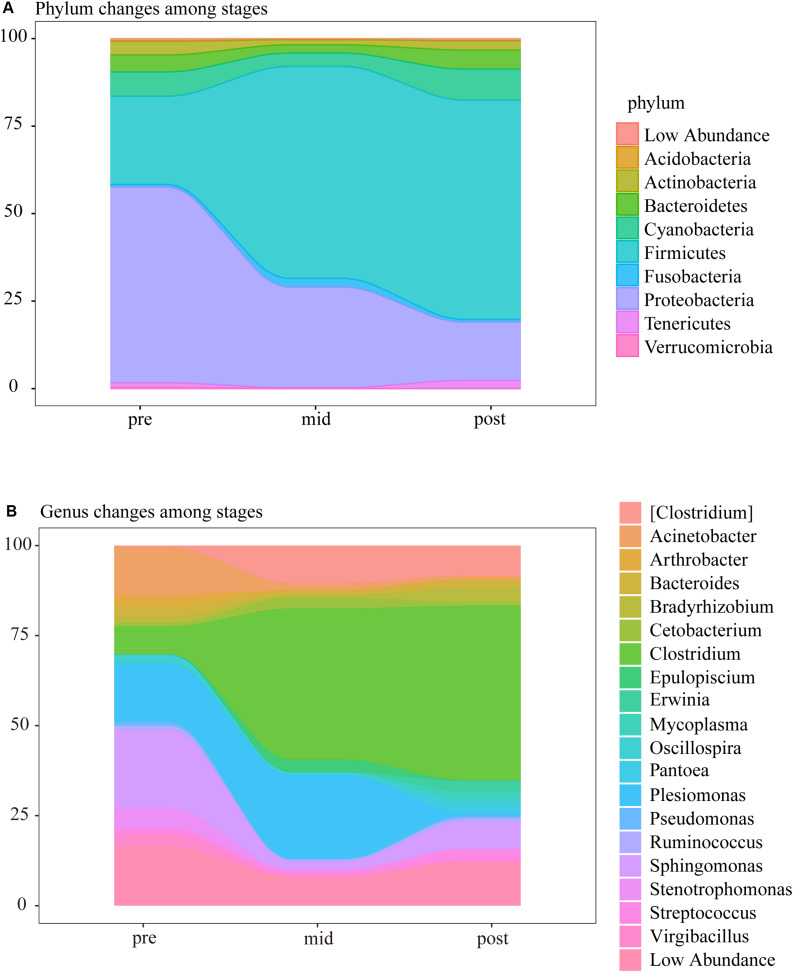
Changes at the **(A)** phylum and **(B)** genus-level composition of intestinal microbial communities during the three weaning stages.

In the three weaning stages, the intestinal microbes were mainly composed of Firmicutes, Proteobacteria, Cyanobacteria, Bacteroidetes, Actinobacteria, Tenericutes, and Fusobacteria, in which the relative abundances of OTUs corresponding to Proteobacteria, Firmicutes, Cyanobacteria, Bacteroides, and Actinomycetes accounted for more than 90% of the total OTUs of the samples from each stage. It was notable that the abundance of Proteobacteria decreased from 56.32% in the pre-weaning stage to 28.91 and 16.84% in the mid and post-weaning stage, while the abundance of Firmicutes increased from 25.29% to 60.70% and 62.81% (Figure 4A and Supplementary Table S5). It was found at the genus level that *Clostridium* of Firmicutes significantly increased in the mid and post-weaning stages compared to the pre-weaning stage, and that the significant decrease in abundance by the post-weaning stage was mainly Proteobacteria, including *Plesiomonas*, *Sphingomonas*, *Acinetobacter*, and *Stenotrophomonas* (Figure 4B and Supplementary Table S6).

#### Intestinal Digestive Enzymes

**FIGURE 5:**
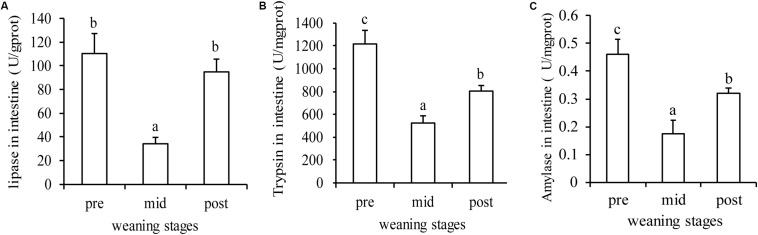
Digestive enzymes changes in during the three stages. **(A)**, **(B)**, and **(C)** are lipase, trypsin and amylase activity in intestine, respectively. Letters, a, b, and c, indicate significant differences between groups (*P* < 0.05) as determined by Duncan’s multiple range test. Values are means ± SE, *n* = 4.

As shown in Figure 5, the three important digestive enzymes showed a significant decrease in the mid-weaning stage compared with the pre-weaning stage, but recovered somewhat in the post-weaning stage (*P* < 0.05). In the post-weaning stage, the intestinal lipase almost returned to the level before weaning (Figure 5A), while the activity of trypsin and amylase was still lower than that in pre-weaning stage (Figures 5B,C).

### Correlation Analysis Between Host and Microbiome

**FIGURE 6:**
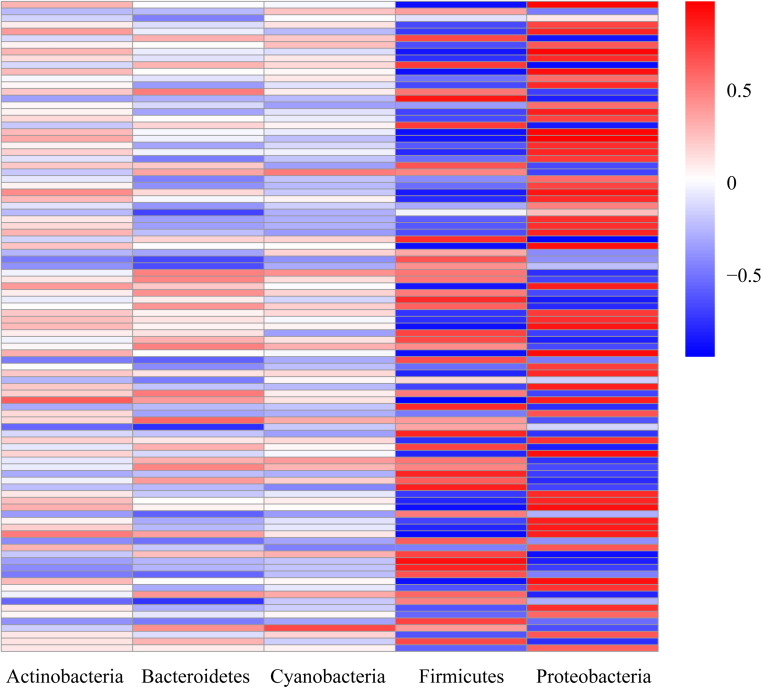
Heatmap of the correlation coefficients. Correlation analysis between the top five intestinal microbes and target genes from the amino acid and lipid metabolism pathways. Correlation coefficients with absolute values greater than 0.6 were considered strongly correlated.

To further test the correlation between host and microbiome, we compared the gut microbiome structure and the liver transcriptome. We correlated the top five principal components (PCs) of the microbiome to the genes of the amino acid and lipid metabolism pathways using the OmicShare ICA tools, which showed that the two major changing microbe (Proteobacteria and Firmicutes) and the target genes were highly correlated (Figure 6).

## Discussion

### Liver Transcriptomic Profile During Weaning

The liver is central to metabolism in fish. Indeed, the transcriptomic results of this study showed that the significant changes in the liver during weaning were largely related to metabolism. Similar changes have been observed during the weaning of mammalian pigs and sheep, which showed different metabolic characteristics before and after weaning (Liu et al., 2016; Wang et al., 2016). It is well known that fish eat different foods, affecting different metabolic characteristics and immune abilities (Li et al., 2017; Yang et al., 2019). As a typical vertebrate herbivore, the grass carp likewise undergoes an extreme transformation from carnivory to herbivory, they experience increased appetite, a resetting of circadian phase, and enhanced digestion and metabolism (Sun et al., 2015). These are all examples of different animals adapting themselves to the challenges of weaning or food habit transition. In this study, the expression of genes related to fatty acid metabolism pathways significantly increased during the weaning process, while the expression of genes related to amino acid metabolism decreased (Figure 2). Before weaning, the main feed included the zooplankton and *Chironomus plumosus*, which were ideal natural baits for larval largemouth bass for its nutrients enriching a lot of protein. Compared with lipids and carbohydrates, proteins would be considered the highest priority utilization among the three major nutrients in fish (Richard, 1991). However, puffed feed inevitably incorporated nutrients such as starch and oil for binders. These mostly plant-derived nutrients may lead to enhanced metabolism of fatty acid-related pathways in the liver of largemouth bass. In the mid-weaning period, the palatability of artificial compound feed was inferior to animal bait such as *Chironomus plumosus*, and we actually found that the big fish had already eaten the little fish. Therefore, forcing the larvae to consume artificial compound feed may have a stressful effect. Our results also found that highly expressed genes in the mid-weaning period were enriched in environmental signal processing pathways (Figure 2), helping the larvae choose food. Furthermore, the anorexic gene leptin was also more highly expressed in the mid-weaning stage than in the other two stages (Cowley et al., 2001) (Supplementary Data Sheet S1). In addition, the low-expression genes in the mid-weaning period were mainly enriched in immune-related pathways, which may be caused by malnutrition due to poor food intake.

### Succession of Dominant Microbe During Weaning

Aided by next-generation sequencing technologies, research on the microbiota of fish intestinal tracts has drastically increased in the past few years. These microorganisms play an important role in the host’s digestion and absorption, immune regulation and disease occurrence (Ivana et al., 2012; Destanie et al., 2018). In fish, there are several possible sources for the intestinal microbiota, and it is generally believed that the processes of bacterial colonization in the early developing fish larvae are complex and depend upon the microbiota found in the: (i) eggs, (ii) larval rearing water, and (iii) the diet (Bolnick et al., 2014; Wang et al., 2018; Yang et al., 2018). Among the many influencing factors, diets would have the strongest effect on intestinal microbes (Wang et al., 2018). In this study, the dominant phylum of intestinal microbes gradually changed from Proteobacteria to Firmicutes with the intake of compound diets during the weaning stage (Figure 4). As found in rainbow trout studies, Firmicutes dominated the gut of fish fed plant source oils, while Proteobacteria was the dominant phyla in fish fed fish source oils (Desai et al., 2012) which is likely due to the addition of plant material in the feed. Another noteworthy finding (Figure 4 and Supplementary Table S5) was that the ratio of intestinal Firmicutes to Bacteroidetes increased in the mid and post-weaning stages, which has also been found in previous studies, where the change reflected fat accumulation or obesity in the host (Zhao, 2013). Studies in sterile mice have shown that gut microbes can promote intestinal absorption of monosaccharides, thereby inducing liver fat production and increasing body fat (Gordon et al., 2004). A high-fat diet could reduce the number of Bacteroidetes bacteria in the intestine of mice, increase the number of Firmicutes and Proteobacteria, and at the same time, the high-fat diet also reduced the number of *Bifidobacteria* in feces and increased the blood fat Polysaccharide (LPS) concentration (Cani et al., 2008). Therefore, the results of gut microbes were contacted with the enhanced liver lipid metabolism pathways we observed, and agreed with the fats in the liver in the actual production of largemouth bass.

In order to strengthen the connection between changes in intestinal microbes and those of the host, the DEGs of the amino acid and lipid metabolism pathways were selected for correlation analysis with the top five microbial phyla. It was found that the two major microbes (Firmicutes and Proteobacteria) that changed in the gut had a strong correlation with the host amino acid and lipid metabolism pathways, which was similar to observations in tilapia under cold stress where host and microbes were co-modulated its response to temperature (Kokou et al., 2018). In fact, host-microbe interactions are a fundamental part of the hologenome concept, where the microbiome composition may be affected by the physiological status of the host (Brucker and Bordenstein, 2013; Theis et al., 2016; Eugene and Ilana, 2018). Similarly, during the weaning stages, largemouth bass and its gut microbes have shown the ability to adapt together to the pressures of changing food sources (Neuman et al., 2016; Kokou et al., 2019; Tan et al., 2019).

### Intestinal Digestive Enzymes

Early weaning is a critical obstacle that all fish must overcome (Enric et al., 2016). Our results from analyses of the liver transcriptome showed that genes with decreased expression mainly enriched in the pathways related to the immune system, such as complement and coagulation cascades, platelet activation, and the IL-17 signaling pathway. Additionally, three important digestive enzymes (trypsin, lipase, and amylase) were selected to examine the intestinal digestive capacities during weaning and we observed significant decreases in enzyme activities in the mid-weaning stage (*P* < 0.05) (Figure 5). This may be due to the fact that the larvae were in a state of adaptation during the mid-weaning stage, and it was difficult to digest and absorb artificial compound feed, resulting in indigestion and malnutrition. As in the study of cobia (Nguyen et al., 2011) and sea bass (Suzer et al., 2007) compared with the feeding of live bait, the survival rate, growth and development and digestibility of the fry in the group fed with compound feed showed a decrease. It was also found that the timely weaning of southern halibut showed a higher survival rate (Faulk and Holt, 2009). However, in the study of pike perch, no difference was found in the digestibility of the two groups of live prey and compound feed (Hamza et al., 2007). It may be related to the different digestive system and species of fish. Of course, the composition, processing technology, palatability and digestibility of puffed feed also would affect growth of fry and the success rate of weaning. While we see multiple obvious changes in gene expression and enzyme activity, in the future more attention should to be paid to the liver immunity and the intestine health of largemouth bass during weaning.

## Conclusion

Overall, the current study provided a comprehensive understanding of the changing dynamics of the liver metabolic characteristics and intestinal microbial community of largemouth bass during weaning. The results showed that liver lipid metabolism obtained enhancement and intestinal microbes were gradually dominated by Firmicutes with weaning progresses. Largemouth bass larvae were been in a state of reduced digestive capacity and immunity in the mid-weaning period. These data could help future research aiming to gain a deeper understanding of fish weaning and to develop optimal weaning strategies for application in aquaculture.

## Data Availability Statement

Sequencing data have been deposited in the SRA under the accession code PRJNA622714 (liver transcriptome) and PRJNA601124 (intestinal microbes).

## Ethics Statement

The animal study was reviewed and approved by the Institutional Animal Care and Use Committee (IACUC) of the College of Animal Science and Technology of Sichuan Agricultural University, Sichuan, China.

## Author Contributions

SY and QL managed the grants, supervised the laboratory work, and led the design of this study. LZ and KH conceived and designed the research, performed the bioinformatics, and also drafted the manuscript. LZ, KH, JS, JL, LL, and XT participated in the tissue sampling and the molecular experiment. All the authors read and approved the final manuscript.

## Conflict of Interest

The authors declare that the research was conducted in the absence of any commercial or financial relationships that could be construed as a potential conflict of interest.

## Footnotes

1 https://github.com/trinityrnaseq/trinityrnaseq/wiki

2 http://www.omicshare.com/tools/Home/Soft/ica

## Supplementary Material

FIGURE S1 Transcriptome and quantitative real-time PCR (qRT-PCR) analyses of the expression of 9 DEGs. Significant differences among the three stages are indicated by different letters (*P* < 0.05). Vertical bars represent means ± SE (*n* = 3).

DATA SHEET S1 The list of differentially expressed genes.
