# Supplementary figures and images for: Co-modulation of Liver Genes and Intestinal Microbiome of Largemouth Bass Larvae (Micropterus salmoides) During Weaning
Source: Front Microbiol. 2020 Jun 17;11:1332. doi: 10.3389/fmicb.2020.01332 (PMC7311569; doi:10.3389/fmicb.2020.01332)

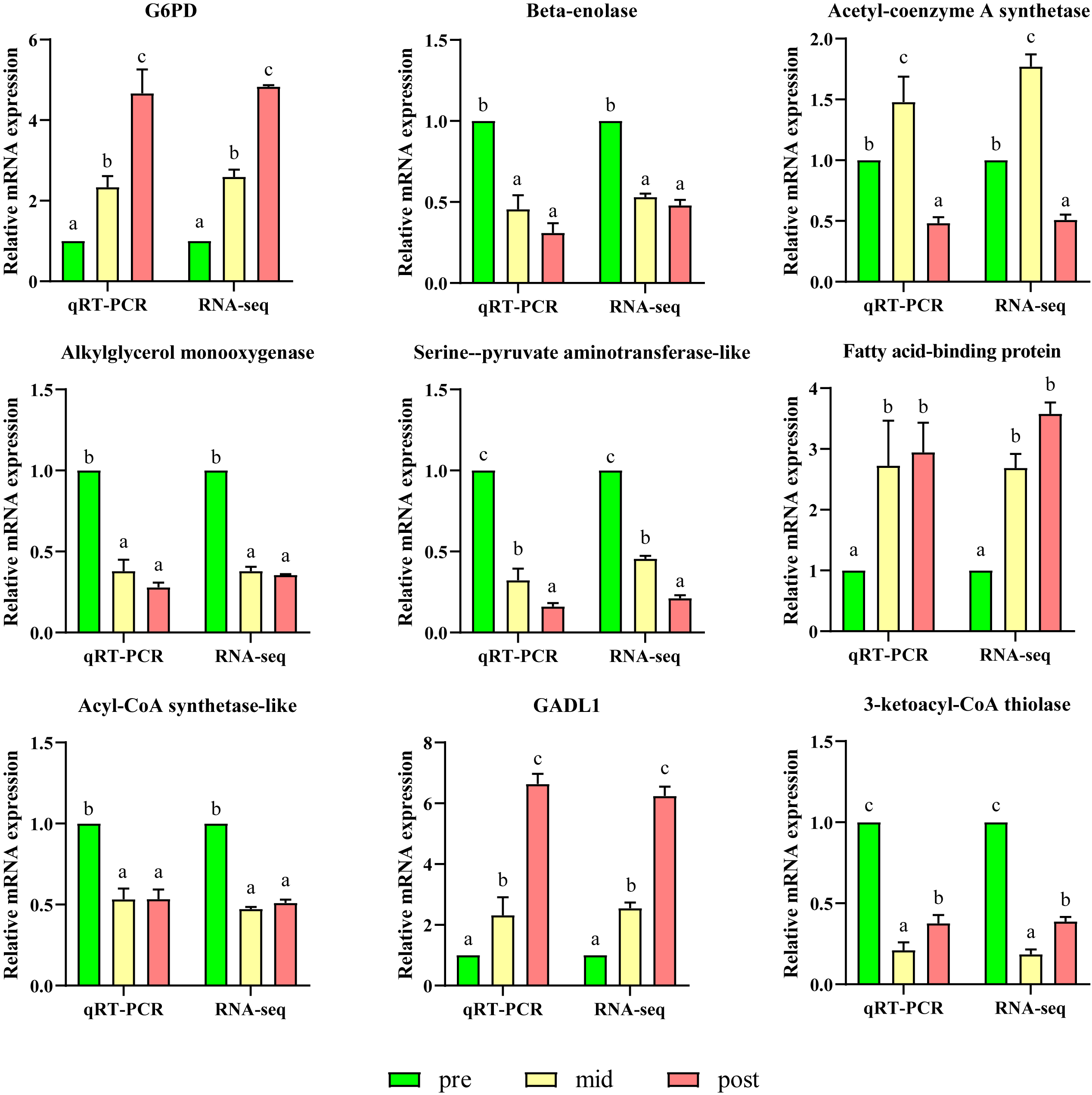

Supplement: FIGURE S1 — Transcriptome and quantitative real-time PCR (qRT-PCR) analyses of the expression of 9 DEGs. Significant differences among the three stages are indicated by different letters (P < 0.05). Vertical bars represent means ± SE (n = 3). [file Image_1.tif]
