# Supplementary material for: Co-modulation of Liver Genes and Intestinal Microbiome of Largemouth Bass Larvae (Micropterus salmoides) During Weaning
Source: Front Microbiol. 2020 Jun 17;11:1332. doi: 10.3389/fmicb.2020.01332 (PMC7311569; doi:10.3389/fmicb.2020.01332)
Supplement: Supplementary file 3 [file Table_1.DOCX]

**Table S1 The Primers for confirming the RNA-seq data by qRT-PCR.**

| Name | Primer sequence (5’–3’) | Amplicon size (bp) |
| --- | --- | --- |
| G6PD | F: CACATTCTCTCTCTGCCCG  R: CCAGCCTTTTGTGCTCATAC | 123 |
| Beta-enolase | F: CATACTTCCTGTTCCTGCCTT  R: TTTACCGTACTTCGCCTTGA | 178 |
| Acetyl-coenzyme A synthetase | F: GTGTCACTAAATGCCTTGTCG  R: ACATCACACTTTGCGTCCC | 100 |
| Alkylglycerol monooxygenase | F: GGGCGTGGACTTCTGCTACT  R: ACTGCTGGGTGAGGGACTGT | 137 |
| Serine--pyruvate aminotransferase-like | F: AGTGTCTGGATTCTTTGCCC  R: GCCACCTCCTTGTGTTTCTT | 90 |
| Fatty acid-binding protein | F: GGTTGATGCTTTCTGCGG  R: CGGTTTAGTGACATTGCCC | 115 |
| Acyl-CoA synthetase-like | F: CACGAGAGGCTATGGATGTCA  R: CACTGTGGTAAAGAGGCAGGTAA | 94 |
| GADL1 | F: GCTTTACTGGGCATCGGCA  R: GCACGGCACCCTCACTTTT | 118 |
| 3-ketoacyl-CoA thiolase | F: CCATCGGACATCCTCTCGGT  R: TACTTGCCTCCTCGTCGCCT | 82 |
| β-act | F: GTATTGTCATGGACTCTGGTG  R: ACGTACGATTTCACGCTCAGC | 182 |
