# Supplementary material for: Co-modulation of Liver Genes and Intestinal Microbiome of Largemouth Bass Larvae (Micropterus salmoides) During Weaning
Source: Front Microbiol. 2020 Jun 17;11:1332. doi: 10.3389/fmicb.2020.01332 (PMC7311569; doi:10.3389/fmicb.2020.01332)
Supplement: Supplementary file 4 [file Table_2.DOCX]

**Table S2. Filtered Reads quality statistics**

| Sample | Total Raw Reads (M) | Total Clean Reads (M) | Total Clean Bases (Gb) | Clean Reads Q20 (%) | Clean Reads Q30 (%) | Clean Reads Ratio (%) |
| --- | --- | --- | --- | --- | --- | --- |
| pre_1 | 71.04 | 67.49 | 6.75 | 97.04 | 88.67 | 95.01 |
| pre_2 | 61.57 | 58.4 | 5.84 | 97.28 | 89.41 | 94.84 |
| pre_3 | 68.78 | 64.98 | 6.5 | 97.12 | 88.95 | 94.48 |
| mid_1 | 77.7 | 71.86 | 7.19 | 97.76 | 90.5 | 92.48 |
| mid_2 | 60.59 | 57.62 | 5.76 | 97.07 | 88.69 | 95.1 |
| mid_3 | 72.7 | 69.04 | 6.9 | 97.09 | 88.86 | 94.97 |
| post_1 | 70 | 67.16 | 6.72 | 97.45 | 89.86 | 95.95 |
| post_2 | 72.1 | 68.55 | 6.85 | 97.15 | 89.01 | 95.07 |
| post_3 | 72.25 | 68.79 | 6.88 | 97.25 | 89.3 | 95.21 |

**Note: Pre, mid and post: pre-weaning, mid-weaning, post-weaning; (M): million.**
