# Supplementary material for: Co-modulation of Liver Genes and Intestinal Microbiome of Largemouth Bass Larvae (Micropterus salmoides) During Weaning
Source: Front Microbiol. 2020 Jun 17;11:1332. doi: 10.3389/fmicb.2020.01332 (PMC7311569; doi:10.3389/fmicb.2020.01332)
Supplement: Supplementary file 5 [file Table_3.DOCX]

**Table S3. Quality indicators of Unigene (bp)**

| Sample | Total Number | Total Length | Mean Length | N50 | N70 | N90 | GC(%) |
| --- | --- | --- | --- | --- | --- | --- | --- |
| pre_1 | 27838 | 27401242 | 984 | 1867 | 1037 | 362 | 45.67 |
| pre_2 | 26524 | 25581622 | 964 | 1807 | 984 | 361 | 45.3 |
| pre_3 | 26142 | 25479702 | 974 | 1782 | 1016 | 367 | 44.93 |
| mid_1 | 32255 | 34220910 | 1060 | 2057 | 1145 | 391 | 45.77 |
| mid_2 | 27012 | 25846014 | 956 | 1765 | 980 | 359 | 45.23 |
| mid_3 | 31570 | 32576627 | 1031 | 1914 | 1103 | 389 | 45.42 |
| post_1 | 28517 | 27901111 | 978 | 1743 | 1018 | 377 | 44.95 |
| post_2 | 30647 | 31251429 | 1019 | 1876 | 1084 | 388 | 45.18 |
| post_3 | 27876 | 28338118 | 1016 | 1802 | 1077 | 395 | 44.79 |
| All-Unigene | 42631 | 62787387 | 1472 | 2659 | 1761 | 653 | 45.03 |
