# Supplementary material for: Co-modulation of Liver Genes and Intestinal Microbiome of Largemouth Bass Larvae (Micropterus salmoides) During Weaning
Source: Front Microbiol. 2020 Jun 17;11:1332. doi: 10.3389/fmicb.2020.01332 (PMC7311569; doi:10.3389/fmicb.2020.01332)
Supplement: Supplementary file 6 [file Table_4.DOCX]

**Table S4. The main enrichment pathways in three weaning stages**

|  | KEGG_A_class | Pathway | Pvalue | Qvalue |
| --- | --- | --- | --- | --- |
| HEGs in pre-weaning stage | Organismal Systems | Cholesterol metabolism | 1.85E-06 | 0.000269 |
|  | Organismal Systems | Antigen processing and presentation | 4.57E-06 | 0.000331 |
|  | Organismal Systems | Vitamin digestion and absorption | 1.93E-05 | 0.000933 |
|  | Metabolism | Cysteine and methionine metabolism | 0.000153 | 0.004446 |
|  | Metabolism | Glycine, serine and threonine metabolism | 0.000257 | 0.006214 |
|  | Metabolism | Arginine and proline metabolism | 0.000938 | 0.018829 |
| LEGs in pre-weaning stage | Organismal Systems | Protein digestion and absorption | 2.79E-26 | 5.86E-24 |
|  | Organismal Systems | Pancreatic secretion | 5.83E-19 | 6.13E-17 |
|  | Metabolism | Biosynthesis of unsaturated fatty acids | 0.000524 | 0.021809 |
|  | Organismal Systems | PPAR signaling pathway | 0.0017 | 0.035705 |
|  | Metabolism | Pantothenate and CoA biosynthesis | 0.004155 | 0.064912 |
|  | Metabolism | Fatty acid metabolism | 0.004159 | 0.064912 |
| HEGs in mid-weaning stage | Cellular Processes | Focal adhesion | 1.66E-07 | 1.88E-05 |
|  | Environmental Information Processing | ECM-receptor interaction | 3.45E-06 | 0.00013 |
|  | Environmental Information Processing | PI3K-Akt signaling pathway | 2.76E-05 | 0.000623 |
|  | Organismal Systems | Estrogen signaling pathway | 0.005343 | 0.062047 |
|  | Cellular Processes | Phagosome | 0.005403 | 0.062047 |
|  | Organismal Systems | Protein digestion and absorption | 0.006424 | 0.062047 |
| LEGs in mid-weaning stage | Organismal Systems | Complement and coagulation cascades | 2.19E-06 | 4.83E-05 |
|  | Organismal Systems | Platelet activation | 0.000379 | 0.003075 |
|  | Metabolism | Ascorbate and aldarate metabolism | 0.003736 | 0.020549 |
|  | Organismal Systems | Thyroid hormone synthesis | 0.007813 | 0.034376 |
|  | Metabolism | Inositol phosphate metabolism | 0.012111 | 0.044407 |
|  | Organismal Systems | IL-17 signaling pathway | 0.127863 | 0.245294 |
| HEGs in post-weaning stage | Organismal Systems | PPAR signaling pathway | 3.88E-08 | 7.8E-06 |
|  | Metabolism | beta-Alanine metabolism | 0.000736 | 0.055578 |
|  | Metabolism | Carbon metabolism | 0.001205 | 0.055578 |
|  | Metabolism | Biosynthesis of unsaturated fatty acids | 0.001311 | 0.055578 |
|  | Metabolism | Fatty acid metabolism | 0.001383 | 0.055578 |
|  | Genetic Information Processing | Proteasome | 0.003498 | 0.100429 |
| LEGs in post-weaning stage | Metabolism | Glycine, serine and threonine metabolism | 1.43E-07 | 2.1E-05 |
|  | Organismal Systems | Complement and coagulation cascades | 1.45E-06 | 0.000102 |
|  | Metabolism | Cysteine and methionine metabolism | 2.08E-06 | 0.000102 |
|  | Metabolism | Metabolic pathways | 4.44E-06 | 0.000163 |
|  | Metabolism | Alanine, aspartate and glutamate metabolism | 0.000314 | 0.007938 |
|  | Metabolism | Biosynthesis of amino acids | 0.000324 | 0.007938 |
