# Supplementary material for: Co-modulation of Liver Genes and Intestinal Microbiome of Largemouth Bass Larvae (Micropterus salmoides) During Weaning
Source: Front Microbiol. 2020 Jun 17;11:1332. doi: 10.3389/fmicb.2020.01332 (PMC7311569; doi:10.3389/fmicb.2020.01332)
Supplement: Supplementary file 7 [file Table_5.DOCX]

**Table S5. Phylum-level** **taxonomic composition of intestinal microbial communities in the three weaning stages.**

| **Phylum** | **% of relative abundance in pre-weaning stage** | **% of relative abundance in mid-weaning stage** | **% of relative abundance in post-weaning stage** |
| --- | --- | --- | --- |
| Firmicutes | 25.29 | 60.70 | 62.81 |
| Proteobacteria | 56.32 | 28.91 | 16.84 |
| Cyanobacteria | 6.96 | 3.82 | 8.87 |
| Bacteroidetes | 4.89 | 2.35 | 5.51 |
| Actinobacteria | 3.87 | 1.45 | 2.69 |
| Tenericutes | 1.35 | 0.21 | 2.28 |
| Fusobacteria | 0.71 | 2.45 | 0.73 |
| Verrucomicrobia | 0.32 | 0.03 | 0.06 |
| Spirochaetes | 0.27 | 0.05 | 0.07 |
