# Supplementary material for: Co-modulation of Liver Genes and Intestinal Microbiome of Largemouth Bass Larvae (Micropterus salmoides) During Weaning
Source: Front Microbiol. 2020 Jun 17;11:1332. doi: 10.3389/fmicb.2020.01332 (PMC7311569; doi:10.3389/fmicb.2020.01332)
Supplement: Supplementary file 8 [file Table_6.DOCX]

**Table S6. Genus-level taxonomic composition of intestinal microbial communities in the three weaning stages.**

| **Phylum** | **Genus** | **% of relative abundance in pre-weaning stage** | **% of relative abundance in mid-weaning stage** | **% of relative abundance in post-weaning stage** |
| --- | --- | --- | --- | --- |
| Firmicutes | *Clostridium* | 6.93 | 36.94 | 41.21 |
|  | *[Clostridium]* | 0.42 | 9.23 | 8.13 |
|  | *Streptococcus* | 1.17 | 1.27 | 2.00 |
|  | *Epulopiscium* | 0.02 | 2.97 | 0.04 |
|  | *Virgibacillus* | 3.00 | 0.10 | 0.01 |
|  | *Oscillospira* | 1.03 | 0.60 | 1.03 |
|  | *Lactobacillus* | 0.44 | 0.32 | 0.68 |
|  | *Ruminococcus* | 0.55 | 0.35 | 0.57 |
| Proteobacteria | *Plesiomonas* | 13.09 | 20.82 | 0.46 |
|  | *Sphingomonas* | 17.87 | 1.85 | 6.10 |
|  | *Acinetobacter* | 10.74 | 1.51 | 0.75 |
|  | *Stenotrophomonas* | 4.39 | 0.22 | 0.38 |
|  | *Bradyrhizobium* | 1.40 | 0.47 | 3.25 |
|  | *Pseudomonas* | 0.73 | 0.22 | 0.21 |
|  | *Enterobacter* | 0.49 | 0.34 | 0.24 |
| Bacteroidetes | *Bacteroides* | 2.00 | 0.86 | 1.40 |
| Fusobacteria | *Cetobacterium* | 0.61 | 2.51 | 0.64 |
| Tenericutes | Mycoplasma | 0.03 | 0.04 | 2.16 |
| Actinobacteria | *Arthrobacter* | 2.28 | 0.31 | 0.20 |
|  | *Bifidobacterium* | 0.61 | 0.40 | 0.34 |
